# Investigating Consumers’ Preference for Acrylamide-Free Cassava Snacks

**DOI:** 10.3390/foods10112721

**Published:** 2021-11-07

**Authors:** Kanokwan Chancharoenchai, Wuthiya Saraithong

**Affiliations:** Faculty of Economics, Kasetsart University, Bangkok 10900, Thailand; kanokwan.c@ku.th

**Keywords:** acrylamide-free, cassava chips, consumers’ preference, marginal willingness to pay, food safety, Thailand

## Abstract

As potato chips are often found to contain a carcinogen, called acrylamide, less-risky chips can alternatively be made from cassava. This study aims at examining consumers’ preference and the factors determining their marginal willingness to pay for acrylamide-free cassava chips. The study is undertaken based on questionnaire surveys with 1077 respondents from all six regions of Thailand. Various socio-economic characteristics, and behavior and perception on relevant issues are included in the OLS estimations of marginal willingness, acting as independent variables. The study finds that people show their preference for acrylamide-free cassava chips, and are willing to pay a premium price of THB 5.86, on average. The results also statistically present, among others, the positive explanatory power of persons’ perception about food safety, especially the dangers of acrylamide, and the size of family on the preference of cassava chips. Adult consumers and those from the northeastern region surprisingly reveal an unfavorable willingness to pay more for non-acrylamide cassava chips. Moreover, the availability of sale promotion can encourage consumers to pay more for healthier cassava chips. The findings should allow producers to understand consumers’ buying behavior and their preference for cassava chips as a substitute product; in turn, this should help to commercialize these products in the wider market.

## 1. Introduction

Thailand’s snacks market has recently grown, with the total value of THB 34,676 million (USD 1 = THB 33.39, as of 21 October 2021) in 2015, increasing from THB 31,498 million in the previous year, of which potato chips account for around 22.7% [1]. The well-liked chips are usually made from potato, which, however, is not Thailand’s native crop. Currently, fresh potato production in the country is not sufficient for domestic consumption, and potatoes need to be imported from such countries as China, Germany, and Canada. In addition, for the high-quality fresh potato required for some food processing, potato seeds have to be imported from the United Kingdom, the Netherlands, and Australia. The value of imports of both fresh potatoes and potato seed has continued to increase from approximately USD 13 million in 2015 to USD 31.9 million in 2019, resulting in not only the high price of fresh and processed potato products, but also the country’s possible trade deficit. In terms of nutrition, although potato is a great source of carbohydrates, it contains glycoalkaloids, which are toxic to humans when consumed in large quantities. Moreover, there is a risk of food contamination from acrylamide, a carcinogen, which was discovered for the first time in 2002 in Sweden, and is theoretically believed to cause cancer in humans [2]. Details on acrylamide can be seen in the work of Kita et al. [3], and the American Cancer Society [4], for example. In later months, various studies confirmed that cooking methods that use high temperature in the process can raise the risk of acrylamide formation [5,6].

This substance is an unscented, transparent, water-soluble solid which is extremely harmful to animals and humans [7]. Studies in various countries have identified the contamination of acrylamide in such popular snacks, as potato chips, bread, and biscuits [8]. This makes it difficult to avoid this substance in producing chips made from potatoes. This finding is also supported by the research of Chu [9], for example, which studies 92 potato snacks from the United Kingdom. Snack brands are found to have acrylamide contamination far above the currently recommended level. At the same time, the U.S. Food and Drug Administration [10] continually monitors the existence of acrylamide in food. Based on the survey, the 2011–2015 data of approximately 2500 individual food product brands show the significant decrease in acrylamide concentration in potato chips, compared to the periodical survey of 2002–2006, which suggests the continued reduction.

In addition to being produced from potatoes, chips can also be produced from cassava, which is one of the most important economic crops in Thailand, with planting areas of approximately 8–9 million rai (1 acre = 2.53 rais). Half of the country’s cassava harvest is in the northeastern region, where a high degree of poverty can be found, compared to other areas [11]. However, in Thailand, in the past, processing food, especially dessert, from fresh cassava was very popular. This is because fresh cassava generally has high content of starch, resulting in astringent taste. If it is used to make cassava chips, it will have a hard texture and a bitter taste. As a result, efforts have been made to improve cassava varieties, focusing on developing the one suitable for consumption, to provide more options for Thai cassava farmers and consumers. The breeding of cassava with a good taste and less starch content could make it suitable for processing fresh produce into edible food, creating added value for the Thai cassava supply chain. Hence, in 2016, a new cassava breeding, Piroon 4, was successfully developed, a cultivar that has been improved to correct its flaws [12]. This new breeding has such qualities as sweet taste, less splintering, and more softness, making it suitable for processing into ready to cook and ready to eat products. As the new variety of fresh cassava has more suitable qualities for direct consumption, this has increased the chance of it being processed into various products, including chips. The major advantage of cassava chips includes the absence of the risk of acrylamide, yet they are not widely and commercially available for consumption in the country.

Cassava chips, an acrylamide-free snack, can be regarded as an alternative to potato chips. However, they are not commonly known, since cassava is often perceived as more suitable for industrial use than for food purposes. Consequently, the question arises concerning their marketable difficulty. To address this difficulty, the investigation of consumers’ behavior could be useful. How people consume snacks depends on their consumption and buying behaviors, which, in turn, rest on their emotional, habitual, and personal traits. Behavior analysis generally involves personal, psychological, and social factors. These factors can, directly and indirectly, influence consumers’ buying decisions of certain products. This decision is considered to be affected by such marketing attributes as product, price, place, and promotion, the so-called four Ps, as well as other environment aspects, such as, economic, political, and cultural factors [13]. Empirically, several works have been undertaken to study consumers’ buying decisions. Susilawati et al. [14] investigate the case of the purchase of snack food products in Indonesia. They find people’s behavior to be influenced by the stimuli factors of the marketing mix concept, which are the attributes of products such as product quality, price, brand, service, and warranties.

Nørgaard et al. [15] tested the purchase intent for healthy snack products of 600 Danish teenagers aged 9–16 years. Price consciousness, health awareness, snack phobia, influence of peers, social activities, and word of mouth were the potential determinants of their demand for innovative snack products. Meanwhile, Damen et al. [16] explored mothers’ buying decisions of snack food for children aged 2–7 years old in Italy. They found that northern Italian mothers express more health concerns compared to those in the south, but the brand is more important to southern Italian mothers.

Moreover, Dinushika and De Silva [17] investigated consumers’ characteristics, their buying behavior, and their perceptions of local market chips. Most of them buy chips due to their taste and brand. At the same time, 46% are fully aware of the various chip brands on the market. The Wilcoxon label rankings test also founds that all factors, such as reliable brand names, frame characteristics, air tightness, price, good quality, overall consumption, taste, and new products, are significant. Their finding seems to align with the work of Kongstad and Giacalone [18], that studies how information labeling determines consumer perceptions about different salt reduction patterns for processed potato products. The results show that sodium can be cut, while keeping the same taste and a clear labeling. However, promotional programs, availability, and packaging colors do not demonstrate a significant explanation, unlike the work of Susilawati et al. [14]. This points to the inconclusive results which, of course, the marketing mix factors of which are worth investigating.

McFadden and Huffman [19] highlight the role of food labels and information in assessing consumers’ food safety valuation. This study applies an alternative approach, called the experimental method, of measuring the preference of adult consumers from three remote regions of the United States after receiving information on the safety risks and advantages of the newly invented biotech potato products. The finding indicates a significant reduction of willingness to pay for traditional products. However, this alternative method has the limitation of time and scale of surveyed respondents. The surveyed questionnaire could then circumvent those limitations.

On consumers’ preference for food containing acrylamide, Harkness and Areal [20] study people’s willingness to pay for a decrease in the amount of acrylamide in baby food in the United Kingdom. They employ a discreet choice experiment to examine such attributes as packaging, production method, acrylamide level, sugar level, and price. The estimation is analyzed by a mixed logit model. They find that, among those attributes, consumers are willing to pay highest price for baby food containing less acrylamide.

As stated above, the scientific innovation of new cassava breeding highlights its importance in terms of not only economic product, but also alternative chips free of acrylamide contamination. However, the market accessibility of cassava chips is still questionable, and needs to be addressed. This study, therefore, aims at examining the factors influencing consumers’ preference and their impact on buyers’ willingness to pay more for the hypothetical non-acrylamide cassava chips. The findings from this investigation would be a useful insight for entrepreneurs and policymakers to draw the proactive strategies, and further contribute to this academic area.

This study’s main hypothesis is that having knowledge about food quality, food safety (especially Acrylamide and its risks), and being young should encourage people to pay higher prices for acrylamide-free cassava chips. Some socio-economic characteristics should also influence individuals’ marginal willingness to pay for cassava chips. The detail of the expected direction of their relationship will be explained later.

To achieve the objective of this study, the paper is structured as follows. The next section describes materials and methods employed in this study. Section three presents the findings, and Section four discusses relevant issues. The last section provides the conclusion of the work.

## 2. Materials and Methods

The study was conducted in accordance with the Declaration of Helsinki, and the protocol was approved by the Kasetsart University Research Ethics Committee (Certificate of Approval No. COA 64/026). First, this section presents the study’s survey design, explaining how the information used is collected. Then, the characteristics of survey sample are provided. Finally, the last part of this section describes the methodology employed.

### 2.1. Survey Design

Surveyed individuals aged 13 years old and older were voluntarily and randomly asked to complete the questionnaires. All subjects gave their informed consent for inclusion before they participated in the study. The questionnaires were developed to elicit individuals’ information. The sentiment of Thai nationals and the concerns for the attributes of food standard and safety are also included, as suggested by Nørgaard et al. [15], Kongstad and Giacalone [18], and Dinushika and De Silva [17]. Respondents were asked to evaluate these issues on a five-point Likert scale ranging from 1 to 5. More positive answers were assigned to the higher values (e.g., 1 = not agreed at all and 5 = extremely agreed). To serve the purpose of addressing how Thai people are willing to pay more for acrylamide-free cassava chips, the questionnaires were thus designed to inquire surveyed individuals and to draw their different preferences for the product. It must be noted that this marginal willingness to pay is a self-measurement designed to retrieve people’s preference under the concept of contingent method applications [21]. The contingent value method is the approach applied to undertake the hypothetical market. This method is properly applied when the product has not been placed in the market, or it has intangible features. As stated above, neither cassava chips themselves nor the fact that acrylamide originates from a chemical reaction after cooking at high heat and can contaminate potato chips are widely known. Consequently, the contingent value method can be reasonably used as the conceptual framework for valuing acrylamide-free cassava chips. The questions used to account for preferences are structured as both the yes-no question (willing or not willing to pay more), and open-ended questions to ask individuals to respond in monetary terms (Thai baht).

Additionally, demographic information, understanding and perception concerning the food (snack) standard, quality, safety, and acrylamide, and buying behavior are also covered in the questionnaire, since these aspects can reveal consumers’ preference on cassava chips free from acrylamide, as suggested by Damen et al. [16], Saraithong [22], and Chancharoenchai [23]. To retrieve this information, the surveyed individuals were asked to answer the multiple-choice questions. Before collecting relevant information from 1077 Thai respondents living in six regions in May–June 2021, the pilot questionnaire was tested with 51 participants to assess its reliability. Cronbach [24] and Nunnaly [25] suggest that Cronbach’s alpha provides the index of reliability test of the questions asked, and the acceptable level is at the conventional level of 70%. The reliability test shows that the Cronbach’s alpha coefficient of the overall surveyed questionnaires is above the conventional level, which implies reliable questions with consistent responses.

### 2.2. Sample Characteristics

Preliminary statistics of 1077 surveyed individuals, shown in Table 1, report that more than half of them were female, and only 31 respondents (2.88%) declared an alternative gender. Meanwhile, most of them were in adulthood or more than 20 years of age, covering the age groups of 20–29 (32.68%), 30–39 (31.57%), and above 39 (23.03%) years old, which is consistent with the population structure of Thailand [26]. Respondents who hold at least a bachelor’s degree were in the majority of the surveyed population, with 903 persons (83.84%) in this category. A similar percentage of respondents was represented by an income of less than THB 15,000 (36.03%), and above THB 25,000 (35.09%). Over half of them were employees in either private (24.79%) or government (27.39%) sectors, while 267 respondents (24.79%) were still studying. Respondents came from all six regions of the country, providing a well-rounded representation of the Thai population in general. One-fourth of the surveyed population came from the central region, where the country’s highest population density can be found [26].

### 2.3. Empirical Methodology

As stated above regarding the importance of cassava in Thailand, and the acrylamide contamination from chemical reaction in the production of potato chips, the rising question of how to make acrylamide-free cassava chips marketable captures the attention of this research. Understanding the details of consumers’ behavior, at least in the Thai case, would be the first step of providing the useful information to policymakers and entrepreneurs for the formulation of appropriate strategies for turning Thai cassava chips into a marketable product. The simple Ordinary Least Square (OLS) method is, hence, applied in order to assess the explanatory power of relevant variables on consumers’ preference as self-reported marginal willingness to pay for acrylamide-free cassava chips (MWTP). This MWTP is the premium price that a surveyed individual is willing to pay for acrylamide-free cassava chips, in addition to the price of traditional potato chips sold in the Thai market. The variables that can hypothetically explain the MWTP are divided into two main groups: the socio-economic (PIF), and behavioral and perception information (BEVP). To assess the influencing characteristics of surveyed individuals, the range of age, gender, occupation, and income are sub-categorized as also shown in Table 2.

The OLS model associated with consumers’ preference conceptual framework is applied to explore the sources of marginal willingness to pay for acrylamide-free products in the case of cassava chips. The parameter of each independent variable, shown in Table 2, is estimated to assess its impact on the marginal willingness to pay for acrylamide-free cassava chips (MWTP). The regression model is expressed in the form of OLS as:

$$MWTP_{i}=\alpha +\overset{P}{\underset{p=1}{\sum }}\overset{n}{\underset{i=1}{\sum }}\beta_{p}PIF_{p,i}+\overset{B}{\underset{b=1}{\sum }}\overset{n}{\underset{i=1}{\sum }}\gamma_{b}BEV_{b,i}+\varepsilon_{i}$$

where the subscript p and b are the explanatory variables of personal information (PIF), and behavior and perception (BEVP), respectively, as stated in Table 2, consisting of 18 variables in the MWTP model. In this study, the sample population, i, covers six regions in Thailand, with 1077 respondents, *n* = 1, 2, 3, …, 1077. The interested effects with respect to those of 18 variables are represented by the parameters, *β_p_* and *γ_b_*. Additionally, the test statistics for the significant explanation of each variable in the above equation are under the null hypothesis of the effect, or *β_p_* and *γ_b_*, indifferent from zero with asymptotic to *t*-statistic at a conventional level of 10% significance level (α = 0.10), or 90% confidence level.

Moreover, the estimations are designed to test for the predicted robustness of model specifications and relevant regressors. Before reporting the estimated ones, several models which included or excluded particular explanatory variables were re-estimated to look for the consistent effect by justifying the sign, magnitude, and significance of those variables included in the models. Moreover, various statistics, for example adjusted-R2 and log-likelihood function, were also applied and compared among estimations to select the models that could accurately explain the MWTP. Therefore, the estimated models are established to capture the ability of variables in explaining the marginal willingness to pay. This could show their strength as the determinants of consumers’ purchasing of acrylamide-free cassava chips.

## 3. Empirical Results

The first part of the study’s empirical results explains respondents’ purchasing behaviors, showing some basic statistics. The next part presents the pairwise comparison of determining factors of marginal willingness to pay. Finally, the last part reports on the estimated results of the determinants of the marginal willingness to pay for acrylamide-free cassava chips.

### 3.1. Buying Behavior

Table 3 provides the primary information on the buying behavior of surveyed respondents. According to the descriptive statistics, the smallest group among them buys snack foods every day. Approximately 30% of respondents, at the same time, buy snack foods twice a week, and around 20% buy snack foods once a week or a month. Mostly, they buy snack for themselves (70.10%) or family (44.57%). Only 19.31% buy snacks for themselves and family, and around 18.76% is for others such as friends, relatives, and so forth. More than 60% of the surveyed population usually buy two to three packs of snack foods each time, and approximately a quarter of them buy only one pack at a time. Not surprisingly, almost 90% are willing to pay a premium price for acrylamide-free cassava chips. Meanwhile, the remaining 12.91% show an unwillingness to pay a premium price.

Those 139 of the 1077 respondents who showed their unwillingness to pay premium prices on acrylamide-free cassava chips, appearing in Table 3, are asked to provide a reason for their decision, as shown in Table 4. Interestingly, 52 respondents (37.41%) somewhat show a protest preference bias. In other words, they do not show their true preference on this issue. Even if they do understand or know about the danger of acrylamide contamination, they still think that the government should take all responsibilities for people’ health, and prevent any cause of cancer. Consequently, they protest their own preference on acrylamide-free content. These respondents were excluded from the surveyed sample to eliminate the estimating problem resulting from possible bias estimators.

With the exclusion of 52 protest preference cases, the further analysis thus covers 1025 respondents who show their true preference for acrylamide-free cassava chips. It is found that the average amount of premium price they are willing to pay for these chips is THB 5.86. This finding provides the evidence that most Thai consumers, at least among the surveyed population, are aware of acrylamide contamination in potato chips, and would prefer the alternative free of this substance. This result is also consistent with Harkness and Areal [20], who demonstrate that consumers in the United Kingdom are willing to pay higher prices for baby food with low acrylamide content.

### 3.2. Pair Wise Comparison of Determining Factors

Before reporting the empirical evidence from the OLS estimation, the ANOVA tests were applied to relate the mean values of willingness to pay premium prices with the respondents’ socio-economic characteristics in order to preliminarily understand their behavior and related aspects. To make a pairwise comparison, the sample was divided into two sub-groups: (1) female (GENF) as group i, and (2) male and alternative gender (otherwise) as group j. The results of the paired sample *t*-test are illustrated in Table 5. The *t*-statistic is tested at a conventional level of a 5% significance level (α = 0.05).

According to the results of paired sample *t*-test, among 12 paired comparisons, seven of them are statistically significant, i.e., the case of education, income, occupation, frequency of buying snack foods, hometown region, the number of family members, and the perception on acrylamide. The group of different genders and age ranges, in contrast, seems to equally value their preference for acrylamide-free cassava chips. The groups of the surveyed population who hold higher than a bachelor’s degree and have an income of more than THB 15,000 per month present statistically and significantly less marginal willingness to pay premium prices compared to other groups. Additionally, the *t*-test also indicates that the group of students is averagely willing to pay more than the others. Meanwhile, the surveyed population who frequently buy snack foods for themselves and their family every day significantly value their preference for acrylamide-free cassava chips higher than the group with less frequency. Knowing that the northeastern region is the area in which the most cassava is cultivated in Thailand, persons from this region should support and favor acrylamide-free cassava chips. However, respondents whose hometown is in the northeastern region have a significantly lower mean of willingness to pay this premium price than other regions. This finding contradicts the common belief. The number of family members demonstrates the significant factor for mean of willingness to pay, as families with more than 4 persons significantly favor cassava chips free of acrylamide content. Moreover, the test results show an indifferent mean of marginal willingness to pay between the group of respondents who have good and less accessibility to information about the standards and food safety of snack foods and chips. On the other hand, individuals who receive clear information about acrylamide are surprisingly willing to pay a lower premium price compared to those who have less information.

### 3.3. Estimated Results

From the nine estimated models, among 18 independent variables, statistically significant factors are identified and presented in Table 6. The explanatory power of respondents’ age, as implied by their generation, is quite strong. This group of variables (AGEBXY and AGEZ) is statistically significant in seven out of the total nine models. The negative significance of AGEBXY in the five models reveals that people aged over 20 years old are less likely to pay extra for cassava chips. Meanwhile, the positive relationship between AGEZ and respondents’ marginal willingness to pay in the model (4) and (7) suggests that those younger than 20 years of age are enthusiastic to pay more for these chips. Gender and education in all model specifications bear no explanatory power in this study.

NFAM is statistically significant in most models where OCST is placed to represent respondents’ occupation as students. The positive relationship between the number of family members (NFAM) and the willingness to pay extra for acrylamide-free cassava chips indicates that individuals from families larger than four persons are pleased to pay more for these chips. However, in these models, the OCST itself has no explanatory power at all. Interestingly, in model (7) and (8) where OCPG is to represent respondents’ occupation, it is statistically significant with a negative relationship, whereas NFAM is insignificant. Working persons seem to be less keen on acrylamide-free cassava chips, and, thus, unwilling to pay extra money for the products. The negative explanatory power of respondents’ income can be found in model (5) and (6), which show that persons having monthly income of THB 15,000 or more are less likely to pay an additional price for acrylamide-free cassava chips.

The statistical significance of respondents’ awareness of nutrition and food safety issues (BEV) in all nine models displays the positive relationship between this variable and their marginal willingness to pay for acrylamide-free cassava chips. More recognition on these issues can lead to people’s readiness to pay more for the chips. The positive explanatory power of respondents’ understanding about acrylamide in food (PERC2), found in all models, indicates that specific food safety knowledge can encourage consumers to pay a further amount for safer snacks. The positive significance of respondents’ considerations of sale promotions (POMO) in all model specifications shows the strong ability of this variable in explaining consumers’ marginal willingness to pay for acrylamide-free cassava chips. Respondents’ hometowns can also influence their buying preference for cassava chips. As it is found in six models, the statistical significance of their domicile in northeastern provinces (NE) shows that they would be less willing to pay a higher price for these chips.

## 4. Discussion

From the results presented above, it can be seen that four variables perform very well in explaining people’s willingness to pay a premium price for acrylamide-free cassava chips. They are BEV, PERC2, NE, and POMO. Persons who strongly attach importance to the issue of nutrition and food safety when buying chips (BEV) will be ready to pay a higher sum for acrylamide-free cassava chips. As they take food safety seriously, they are probably aware of the existence of acrylamide in food, thus spending more to avoid this substance is reasonable for them. This coincides with the significance of PERC2, which indicates that individuals who know about acrylamide and its potential danger are in favor of paying an extra amount in order to avert this harm. The explanatory power of these two variables is complementary. This result is found to be in line with the findings of Saraithong [22], and Chancharoenchai [23], showing that Thai people’s concerns about food safety, food quality, and the potential risks from food contamination can influence their willingness to pay higher prices for specified attributes of food products. This finding thus highlights highly supportive evidence for the incremental consciousness concerning and willingness to pay for food safety and food quality of Thai people.

Furthermore, Dolgopolova and Teuber [27] found that the presence of health benefits in food products increases consumers’ marginal willingness to pay for them. Likewise, knowing about acrylamide and its threats may suggest realizing the benefits of its absence, which could boost people’s marginal willingness to pay. This could become insightful information to entrepreneurs and the government, allowing them to incorporate strategies to enhance food standards and differentiate the market. Aside from the analyses mentioned before, there are also other works studying consumers’ preference, which investigate the statistical significance of health consciousness on buyers’ willingness to pay premium price for healthier food products [28,29,30,31]. The continued interest in this area of research, involving a variety of countries and products, signifies the rising passion in food safety issues at the world level, which in turn indicates a more complex international trading system.

From the above results, basic understanding about the food safety and quality regarding the production of chips and cassava can influence a person’s purchasing behavior by increasing their marginal willingness to pay. Therefore, providing and circulating information that allows the public to develop correct understanding about food safety, especially concerning the threat of acrylamide in food, should be beneficial in terms of both consumers’ welfare and businesses’ profit making. Another message that should be distributed is that concerning new opportunities for farmers from the value creation of cassava products, which could fulfill the hope of improving farmers’ living standard. Equipped with this knowledge, individuals may be encouraged to pay higher prices for chips with less carcinogenic risk, made from cassava. The statistical significance of people’s understanding about acrylamide in this study suggests the importance of education on food safety through both formal and informal channels. Formal education in schools given to children early on in their lives could build a foundation. At the same time, the short-term provision of information on the development and technological advances in food safety issues, including such a novel food as cassava chips, is also equally important for generating understanding among the public. This requires collaboration between the government and private sectors in order for the message to be received by people.

As for respondents’ domicile, at first glance, it may seem rational to assume that people from the northeastern region (NE) should agree with paying more for acrylamide-free cassava chips, as this part of the country is the most densely cultivated area of cassava. The processing of cassava into chips should generate more opportunities for cassava farmers, and eventually raise their income. However, the empirical evidence surprisingly reveals otherwise. It shows that more than 60% of the respondents from the northeastern region express less concern about nutrition and food safety when buying food products. Moreover, the northeastern region of Thailand is commonly known for having the highest number of people living in poverty, with low gross domestic product compared to other areas. People’s minds may be more on their rising expenditure due to higher prices of cassava chips than on profiting from an increase in benefit from consuming non-acrylamide food. Marketing aspects also carry the strong explanatory power for paying a premium price for acrylamide-free cassava chips. From the findings, with the sale promotion measures in place (POMO), people are eager to pay more for healthier cassava chips. This reflects the necessity of having an appropriate marketing strategy. However, this result is different from the study by Ali and Ali [31], which displays that marketing campaigns are unlikely to persuade persons to pay extra for healthy food.

Another group of variables that can explain people’s willingness to pay a premium price for acrylamide-free cassava chips includes the number of family members (NFAM) and respondents’ age (AGE). The explanatory power of the number of family members (NFAM) indicates that the size of respondents’ households can positively affect their buying preference for cassava chips. The larger the family they come from, the more likely they are to pay a higher price for the chips. It could be explained that a larger family may consist of several members from different generations, including the young and the elderly, who are more vulnerable. With these two groups in the household, the family may be more positively responsive to healthier or less risky food products, thereby willing to pay extra for them. On this issue, Muhammada et al. [32] also find that people with larger household size are likely to pay more for organic products.

In most models, a person’s age can significantly affect their payment for the said cassava chips. The results show that adults are reluctant to pay a higher price for acrylamide-free cassava chips. On the other hand, younger generations tend to be more open minded toward cassava chips. This may be because chips are usually more popular among the young. Moreover, there is a general perception among Thai people that chips are Western snacks, thus older respondents tend not to favor them or pay much for them. The importance of purchasers’ age as the determinant of their willingness to pay is supported by previous studies, for instance, [29,33,34,35]. Additionally, regarding the consumer buying decision process, Kotler [36] suggests that people of different ages will inevitably have different preferences for products. It is more likely that teenagers would spend more on novelty and fashionable goods.

As indicated earlier, people with strong purchasing power, such as adults and working persons, are not keen on paying more for acrylamide-free cassava chips. Combining this with the importance of understanding food safety, room for improvement should be provided by raising awareness among these groups about cassava chips. Once they truly understand the threat of acrylamide in food, backed up by their financial ability, they may be pleased to spend more for acrylamide-free cassava chips. Furthermore, various forms of sale promotion and market communication can play a major role in making these chips more attractive for these big spender individuals.

This study offers an insight into consumers’ preference and marginal willingness to pay for innovative product, acrylamide-free cassava chips. This can be considered its strength as, thus far, there is no such a work on this particular subject. It can be beneficial to both policymakers and practitioners. However, this work has its own limitation. As the population of this study is Thai people aged 13 years old and older, they consist of youth, who tend to have no income of their own, instead relying on allowance from parents or relatives. Consequently, their marginal willingness to pay, which is derived from the self-measurement question, may, somewhat, not precisely reflect their true preference. The application based upon this analysis should be cautioned about this limitation.

## 5. Conclusions

The findings address the research question posed earlier. They provide the missing link that should allow producers to understand consumers’ buying behaviors and the factors influencing their preference for cassava chips. With this knowledge, new cassava breeding can be commercially transformed into a healthy product responding to the global concerns on food safety.

According to the findings explained previously, consumers’ buying preferences and their marginal willingness to pay for acrylamide-free cassava chips are determined by various factors. Cassava chips are novel in their nature, consequently, ensuring their constant development can be quite complex. Since the production of cassava chips relies very much on the new breeding of fresh produce, their success requires for farmers to get uninterrupted access to this particular cassava stake. Government agencies, private enterprises, and education institutions need to secure the continual flow of this cassava stake for farmers.

From the new breeding patent to an innovated product, for cassava chips to succeed in the market, they need to be promoted by producers and accepted more widely among consumers so that the benefit can be reaped both at micro and macro levels. At the micro level, consumers can enjoy healthier snacks, and producers of cassava chips can enhance their market. On the macro front, cassava farmers can make more money from new processing opportunities of their product. This could help to improve the financial stability of the agricultural sector. Moreover, the substitution of cassava for potato in the production of chips could result in the possible decrease in the imports of potato, thus, the country can save some foreign currency. Additionally, with a higher consumption of acrylamide-free cassava chips, consumers’ health risk decreases, eventually lessening the burden on the government’s health care expenditure. In the long term, the promotion of cassava chips could lead to the sustainable improvement of people’s welfare.

## Figures and Tables

**Table 1:** Socio-economic characteristics of surveyed individuals.

Item		Frequency	Percentage
Total Respondents		1077	-
Gender	Female	641	59.52
	Male	405	37.60
	Alternative	31	2.88
Age	≤20 years old	137	12.72
	20–29 years old	352	32.68
	30–39 years old	340	31.57
	≥40 years old	248	23.03
Education	<bachelor’s degree	174	16.16
	≥bachelor’s degree	903	83.84
Income	≤THB 15,000	388	36.03
	THB 15,001–25,000	311	28.88
	>THB 25,000	378	35.09
Region	North	142	13.18
	Northeast	202	18.76
	West	112	10.40
	Central	275	25.53
	East	142	13.18
	South	204	18.94
Occupation	Student	267	24.79
	Private worker	267	24.79
	Government worker	295	27.39
	Others *	248	23.03

Note: * Surveyed individuals are unemployed, retired, business owners, freelance, or housewives.

**Table 2:** Definition of relevant variables in the estimations.

Variable	Definition	Variable	Definition
MWTP	This dependent variable is the respondents’ marginal willingness to pay (in Thai baht) for cassava chips that have no contamination of acrylamide. It is the incremental amount from the market price of potato chips in Thailand which reflects the preference for acrylamide-free chips made from cassava.		
Personal information (PIF) presents the socio-economic characteristics of surveyed individuals.			
AGE:	Respondent’s age is divided into four groups by generation, which are GenZ, GenY, GenX, and Baby Boomer as follows: AGEBXY: Baby Boomer, GenX, and GenY = 1; otherwise = 0. AGEZ: GenZ = 1; otherwise = 0. AGEBX: Baby Boomer and GenX = 1; otherwise = 0. AGEZY: GenY and GenZ = 1; otherwise = 0.	GEN:	To understand the explanatory power of different genders, this study groups gender variable as follows: GENF: Female = 1; otherwise = 0. GENFA: Female and LGBT = 1; otherwise = 0. GENA: LGBT = 1; otherwise = 0. As females generally has a risk aversion attitude, especially compared to males, they would tend to be more conscientious on health. They would thus be willing to pay more on acrylamide-free chips.
NFAM	Respondents’ number of family members. This factor could affect the MWTP in two directions, through either purchasing power or health concern, depending on which one would dominate. Having more than 4 persons in the family = 1; otherwise = 0.	EDUB	Respondents’ educational level is a dichotomous variable. Education could indicate how well individuals know and understand the importance of the effect of any substance contained in food. Higher education would induce the preference to pay for acrylamide-free chips. Bachelor and higher degrees = 1; otherwise = 0.
OC:	To capture the reflection of attitude concerning acrylamide through social environment and health consciousness, the occupation is thus separated as follows: OCST: student = 1; otherwise = 0.OCPG: employee of public and private organizations = 1; otherwise = 0.	IN:	The interest in the determination of purchasing power is taken by respondents’ monthly income. The monthly income is separated into two ranges as follows: IN15: THB 15,000 and higher = 1; otherwise = 0. This is based upon the average monthly salary of new coming workers with the bachelor’s degree in general. IN25: THB 25,000 and higher = 1; otherwise = 0. This is referred to the average monthly salary of specific job with bachelor’s degree requirement.
NE	The northeastern region is the most cultivated area of cassava in Thailand. Those living in the northeastern region should support cassava products in the belief of the cause of income creation. Respondents’ hometown is denoted as dichotomous variable: northeastern region = 1; otherwise = 0.	CENES	This factor would help prove the hypothesis of wealth effect as it should be positively related to MWTP. Because the central and eastern regions of Thailand have a higher gross domestic product, people who live in these regions would be more willing to pay for better health. Respondents’ current residence is taken as dichotomous variable: central and eastern regions = 1, otherwise = 0.
Behavior and Perception (BEVP) are the variables that measure the behavioral biasedness and attitude of surveyed individuals in particular issues.			
UNDS	Understanding about the standards and safety of chips, presented in percentage form. The higher the percentage, the better the understanding.	PRIDE	Respondents’ national pride is corresponded in the form of five related questions. This presents the Thai national sentiment. The higher level of this sentiment would, thus, reflect the support for acrylamide-free cassava chips.
FREQ	Respondents’ frequency in buying snack. People who buy snack foods more often should be willing to pay more for acrylamide-free cassava chips, with the hope of reducing the risk of cancer. Every day = 1; otherwise = 0.	EFAM	The purpose of respondents’ buying chips for own self and family is marked as 1, otherwise = 0. This factor is presumed to show the positive relationship with MWTP due to the concern for their own health and for the health of family members.
PWTP	Mean of perception level about food and chips without acrylamide before deciding to buy chips. People who perceive more about this information would, of course, be willing to pay for acrylamide-free in the belief of reducing cancer risk.	QWTP	Mean of perception level about chips quality, and food sanitary when deciding to buy chips. The higher mean score presents a higher concern for health, thus the willingness to pay for acrylamide-free chips should also be high.
BEV	Respondents’ consideration of nutrition and food safety. People who have concerns about nutrition and food when buying food products should show their preference for acrylamide-free cassava chips. Very important = 5; not important = 1.	PERC1	Respondents’ access to information about the standards and food safety of snack and chips. Individuals who have more information about those issue present more concerns about what they eat. They would maintain strong preference on acrylamide-free chips, so they would be willing to pay for them. Having information = 1; otherwise = 0.
PERC2	Respondents’ access to information about acrylamide in food. Individuals who access to information about acrylamide content and its possible cancer risk would preferably trade-off for any food product without this substance. Having information = 1; otherwise = 0.	POMO	Respondents’ consideration of sale promotion is presented in the form of the mean of three questions about advertising and distribution channels. If people undertake more of this factor, they should know more about carcinogenic contamination. Consequently, they should be willing to pay more for the carcinogen-free substitute product.

**Table 3:** Buying behavior and marginal willingness to pay for acrylamide-free cassava chips.

Item		Frequency	Percentage
Snack buying frequency	Every day	157	14.58
	Two time per week	324	30.08
	One time per week	224	20.80
	One time per month	202	18.76
	Uncertain	170	15.78
Purpose of buying snack *	Own self	755	70.10
	Family	480	44.57
	Own self and family	208	19.31
	Others	202	18.76
Buying quantity each time	1 snack pack	250	23.21
	2–3 snack pack	668	62.02
	4–5 snack pack	100	9.29
	More than 5 snack packs	59	5.48
Willing to pay	Willing	938	87.09
	Not willing	139	12.91

Note: * Respondents can provide more than one answer on this item.

**Table 4:** Reason for unwillingness to pay premium price on acrylamide-free cassava chips of 139 respondents.

Reasons for Unwillingness to Pay More	Frequency	Percentage
Do not believe it tastes like potato chips.	64	46.04
Do not believe that potato chips contain carcinogen.	13	9.35
Think the amount of acrylamide is too little to cause cancer.	8	5.76
The government should take all responsibilities for people’s health and prevent any cause of cancer.	52	37.41
No comment.	2	1.44

**Table 5:** Paired sample *t*-test on the average determining factors.

Pairwise Comparison: (Group i–Group j)	Mean of Marginal Willingness to Pay (MWTP)							
	Mean Value: MWTP		95% CI of the Mean Difference		*t*-Stat	Test for Null Hypothesis of Mean Difference (Means of Group i Less of Mean of Group j): Sig. Level		
	Group i	Group j	Lower	Upper		<zero	=zero	>zero
Gender: GENF-otherwise	5.92	5.20	−1.61	0.19	−1.55	0.940	0.121	0.063
Age: AGEZY-otherwise	5.71	5.36	−1.40	0.70	−0.66	0.746	0.509	0.254
Education: EDUB-otherwise	5.34	7.23	5.19	6.07	2.61	0.005	0.001	0.995
Income: IN15-otherwise	5.01	6.76	0.78	2.72	3.55	0.000	0.000	0.100
Occupation: OCST-otherwise	8.31	4.75	−4.68	−2.44	−6.26	1.000	0.000	0.000
Frequency: FREQ-otherwise	6.60	5.47	−2.39	0.12	−1.77	0.962	0.076	0.038
Region: NE-otherwise	4.37	6.19	0.72	2.92	3.25	0.001	0.001	0.999
Residence: CENES-otherwise	5.82	5.91	−0.84	1.02	0.12	0.422	0.843	0.578
Family members: NFAM-otherwise	6.54	5.50	−2.05	−0.03	−2.02	0.978	0.044	0.022
Purpose of buying: EFAM-otherwise	5.31	5.98	−0.51	1.85	1.11	0.133	0.266	0.867
Standard Perception: PERC1-otherwise	5.45	5.98	−0.40	1.46	1.12	0.131	0.263	0.868
Acrylamide Perception: PERC2-otherwise	4.76	6.32	0.68	2.45	3.48	0.000	0.001	0.100

Note: CI is confidence interval, *t*-stat is the value of *t*-statistic, sig. level is significant level.

**Table 6:** The empirical results of MWTP in the case of cassava chips.

Variable	Model 1	Model 2	Model 3	Model 4	Model 5	Model 6	Model 7	Model 8	Model 9
Constant term	0.72 (1.87)	0.70 (1.87)	0.81 (1.87)	−3.80 (1.87) *	−1.57 (1.83)	−1.19 (1.92)	2.50 (1.87)	1.48 (1.85)	1.49 (1.85)
AGEZ	-	-	-	4.51 (0.97) *	-	-	3.98 (0.98) *	-	-
AGEZY	-	-	-	-	-	−0.37 (0.59)	-	-	-
AGEBX	-	-	-	-	0.37 (0.59)	-	-	-	-
AGEBXY	−4.51 (0.97) *	−4.52 (0.97) *	−4.55 (0.97) *	-	-	-	-	−3.98 (0.98) *	−3.93 (0.95) *
GENF	0.28 (0.48)	-	-	0.28 (0.48)	0.40 (0.48)	0.40 (0.48)	0.16 (0.47)	0.16 (0.47)	0.17 (0.47)
GENFA	-	0.33 (0.49)	-	-	-	-	-	-	-
GENA	-	-	0.30 (1.39)	-	-	-	-	-	-
EDUB	1.14 (0.81)	1.13 (0.81)	1.19 (0.81)	1.14 (0.81)	−0.85 (0.70)	−0.85 (0.70)	1.20 (0.81)	1.20 (0.81)	1.16 (0.81)
NFAM	0.90 (0.48) *	0.90 (0.48) *	0.89 (0.48) *	0.90 (0.48) *	0.99 (0.49) *	0.99 (0.49) *	0.71 (0.49)	0.71 (0.49)	0.72 (0.49)
OCST	0.22 (0.21)	0.21 (0.21)	0.22 (0.21)	0.22 (0.21)	0.21 (0.21)	0.21 (0.21)	-	-	-
OCPG	-	-	-	-	-	-	−1.89 (0.55) *	−1.89 (0.55) *	−1.88 (0.52)
IN_15	−0.20 (0.56)	−0.20 (0.56)	−0.19 (0.56)	−0.20 (0.56)	−1.06 (0.56) *	−1.06 (0.56) *	0.38 (0.58)	0.38 (0.58)	-
IN_25	-	-	-	-	-	-	-	-	0.62 (0.51)
UNDS	2.17 (1.39)	2.15 (1.39)	2.15 (1.39)	2.17 (1.39)	2.30 (1.40)	2.30 (1.40)	1.61 (1.39)	1.61 (1.39)	1.59 (1.39)
PRIDE	0.00 (0.35)	0.01 (0.35)	−0.01 (0.35)	0.00 (0.35)	−0.26 (0.35)	−0.26 (0.35)	0.06 (0.34)	0.06 (0.34)	0.06 (0.34)
FREQ	0.94 (0.66)	0.93 (0.66)	0.95 (0.66)	0.94 (0.66)	0.99 (0.67)	0.99 (0.67)	0.93 (0.66)	0.93 (0.66)	0.95 (0.66)
EFAM	−0.31 (0.60)	−0.32 (0.60)	−0.32 (0.60)	−0.31 (0.60)	−0.62 (0.61)	−0.62 (0.61)	−0.34 (0.60)	−0.34 (0.60)	−0.35 (0.60)
PWTP1	0.31 (0.48)	0.31 (0.48)	0.31 (0.48)	0.31 (0.48)	0.27 (0.48)	0.27 (0.48)	0.37 (0.47)	0.37 (0.47)	0.34 (0.47)
QWTP1	−0.25 (0.52)	−0.26 (0.52)	−0.24 (0.52)	−0.25 (0.52)	−0.12 (0.53)	−0.12 (0.53)	−0.34 (0.52)	−0.34 (0.52)	−0.34 (0.52)
BEV	0.74 (0.40) *	0.74 (0.40) *	0.75 (0.40) *	0.74 (0.40) *	0.81 (0.40) *	0.81 (0.40) *	0.68 (0.40) *	0.68 (0.40) *	0.67 (0.40) *
PERC1	0.26 (0.53)	0.27 (0.53)	0.26 (0.53)	0.26 (0.53)	0.09 (0.54)	0.09 (0.54)	0.21 (0.53)	0.21 (0.53)	0.19 (0.53)
PERC2	1.21 (0.51) *	1.20 (0.51) *	1.19 (0.51) *	1.21 (0.51) *	1.40 (0.51) *	1.40 (0.51) *	1.08 (0.51) *	1.08 (0.51) *	1.07 (0.50) *
POMO	0.73 (0.33) *	0.73 (0.33) *	0.74 (0.33) *	0.73 (0.33) *	0.93 (0.33) *	0.93 (0.33) *	0.78 (0.32) *	0.78 (0.32) *	0.82 (0.33) *
NE	−1.07 (0.61) *	−1.08 (0.61) *	−1.09 (0.61) *	−1.07 (0.61) *	−1.55 (0.61) *	−1.55 (0.61) *	−1.01 (0.61)	−1.01 (0.61)	−0.99 (0.61)
CENES	0.52 (0.49)	0.51 (0.49)	0.52 (0.49)	0.52 (0.49)	0.21 (0.49)	0.21 (0.49)	0.49 (0.48)	0.49 (0.48)	0.48 (0.48)
Adj. R^2^	0.07	0.07	0.07	0.07	0.05	0.05	0.08	0.08	0.08
Log-likelihood	−3479.3	−3479.2	−3479.4	−3479.3	−3490.0	−3490.0	−3473.7	−3473.7	−3473.2

Note: Adj.R^2^ is denoted adjusted R-square. * is the significant at 10% significant level (α = 0.10). The number in the bracket is sum squared error (SEE).

## Data Availability

Dataset is available upon request.
